# Mutagenesis of the fusion peptide-like domain of hepatitis C virus E1 glycoprotein: involvement in cell fusion and virus entry

**DOI:** 10.1186/1423-0127-16-89

**Published:** 2009-09-24

**Authors:** Hsiao-Fen Li, Chia-Hsuan Huang, Li-Shuang Ai, Chin-Kai Chuang, Steve SL Chen

**Affiliations:** 1 Institute of Biomedical Sciences, Academia Sinica, Taipei 11529, Taiwan; 2 Division of Biotechnology, Animal Technology Institute Taiwan, Miaoli 35053, Taiwan

## Abstract

**Background:**

Envelope (E) glycoprotein E2 of the hepatitis C virus (HCV) mediates binding of the virus to target cell receptors. Nevertheless, the precise role of E1 in viral entry remains elusive.

**Methods:**

To understand the involvement of the fusion peptide-like domain positioned at residues 264 to 290 within envelope glycoprotein E1 in HCV infection, mutants with Ala and Asn substitutions for residues conserved between HCV and E proteins of flaviviruses or the fusion proteins of paramyxoviruses were constructed by site-directed mutagenesis and their effects on membrane fusion and viral infectivity were examined.

**Results:**

None of these mutations affected the synthesis or cell surface expression of envelope proteins, nor did they alter the formation of a non-covalent E1-E2 heterodimer or E2 binding to the large extracellular loop of CD81. The Cys residues located at positions 272 and 281 were unlikely involved in intra- or intermolecular disulfide bond formation. With the exception of the G267A mutant, which showed increased cell fusion, other mutants displayed reduced or marginally inhibited cell fusion capacities compared to the wild-type (WT) E1E2. The G267A mutant was also an exception in human immunodeficiency virus type 1 (HIV-1)/HCV E1E2 pseudotyping analyses, in that it showed higher one-cycle infectivity; all other mutants exhibited greatly or partially reduced viral entry versus the WT pseudotype. All but the G278A and D279N mutants showed a WT-like profile of E1E2 incorporation into HIV-1 particles. Since C272A, C281A, G282A, and G288A pseudotypes bound to Huh7 cells as effectively as did the WT pseudotype, the reduced infectivity of these pseudotypes was due to their ability to inhibit cell fusion.

**Conclusion:**

Our results indicate that specific residues, but not the structure, of this fusion peptide-like domain are required for mediating cell fusion and viral entry.

## Background

The hepatitis C virus (HCV) is an enveloped, positive-stranded RNA virus classified in the *Hepacivirus *genus of the family Flaviviridae with a genome of 9.6 kb. More than six distinct genotypes exist, with genotypes 1a and 1b being the most prevalent worldwide. The viral genome is translated in host cells into a ~3000-amino acid (aa)-long polyprotein, which is cleaved co-translationally and post-translationally by both host signal peptidases and viral proteases to generate several distinct polypeptides, including three structural proteins located at the N-terminal region of the polyprotein, i.e., the core protein and two envelope (E) glycoproteins (E1, polyprotein residues 192 to 383; and E2, residues 384 to 746), the p7 protein, and six nonstructural proteins (NS2, NS3, NS4A, NS4B, NS5A, and NS5B). E1 and E2 form a non-covalent heterodimer [1] and are heavily N-linked glycosylated type I integral transmembrane proteins, with an N-terminal ectodomain and a C-terminal hydrophobic anchor domains (reviewed in [2,3]). E1 and E2 associate to form two types of complexes: (i) heterodimers stabilized by non-covalent bonds, which probably represent the prebudding and functional form of the viral envelope; and (ii) high-molecular-mass disulfide-bonded aggregates, which may represent misfolded proteins [4-7]. Both types of complexes are retained in endoplasmic reticula (ER), the proposed site for HCV assembly and budding. In addition to anchoring to ER, the transmembrane domains of E1 and E2 also play a role in the assembly of an E1E2 heterodimer (reviewed in [8]). E1 and E2 physically interact with each other to play major roles in virus binding and entry into target cells [9-11].

To circumvent the lack of a reliable and robust in vitro system for virus propagation, various pseudotype viruses (HCVpp) expressing HCV E1 and E2 glycoproteins were developed in the past as mimics to study virus-cell interactions. These include vesicular stomatitis virus (VSV) pseudotypes expressing chimeric HCV E1E2 glycoproteins that encode the transmembrane and cytoplasmic domains of VSV glycoprotein G [12-14] and infectious pseudotype particles utilizing retroviral and lentiviral vectors expressing HCV E1E2 proteins [15-24]. A more-recently developed culture system based on an HCV genotype 2a JFH1 RNA clone, isolated from a fulminant hepatitis patient, to produce an infectious virus in cell culture (HCVcc) has tremendously facilitated our understanding of the HCV replication cycle [25-28]. So far, a number of cellular proteins have been identified as candidate entry receptors (reviewed in [29]). These molecules include the tetraspanin protein, CD81 [30,31], scavenger receptor class B type I [32], a cellular protein that binds high-density lipoprotein, the low-density lipoprotein receptor [33], C-type lectins dendritic cell-specific intercellular adhesion molecule grabbing nonintegrin (DC-SIGN) and L-SIGN [34-37], heparin sulfates [38], and the asialoglycoprotein receptor [39]. CD81 was also shown to facilitate HCVcc entry [25-28]. The tight junction proteins, claudin-1, -6, and -9, were also implicated as entry cofactors in HCV infection [40,41]. In addition, occludin was recently shown to play a critical role in viral entry [42,43].

The entry of both HCVpp and HCVcc is mediated by E1 and E2 in a pH-dependent manner [15,21,25-27,44]. After E2 binding to receptors on the host cell surface and receptor-mediated endocytosis, HCV enters target cells via an acid-activated membrane fusion event, where the viral envelope fuses with an endosomal membrane by an as yet unidentified mechanism. This low pH-induced conformational rearrangement in HCV glycoproteins is consistent with a membrane fusion mechanism similar to that reported for glycoproteins of flaviviruses such as tick-borne encephalitis virus [45,46], dengue virus [21,47], and West Nile virus [48,49]. The E protein of flaviviruses participates in binding to cellular receptors and interacts with cell membranes for fusion and subsequent cell entry, as well as in directing viral assembly and budding (reviewed in [50]). The E protein of flaviviruses folds co-translationally with a regulatory protein, termed the prM. This heterodimeric interaction is important for the correct folding and transport of the fusion protein. The prM is then cleaved by the cellular protease, furin, at a late stage in the secretory pathway, which is a critical regulatory step for subsequent fusion [51,52]. In these class II fusion proteins, low pH-triggered conformational changes of glycoproteins lead to transition from a prefusion, dimeric E protein to a fusion-competent β-sheet trimer, in which the fusion loop is exposed to and inserts into target endosomal membranes to initiate the fusion process (reviewed in [53]).

So far, the molecular events leading to membrane fusion and cell entry by HCV are still poorly understood. Similar to alphaviruses and flaviviruses, the organization of HCV E1 and E2 proteins encoded in tandem implies a similar arrangement of a regulatory protein located N-terminal to a fusion protein. Nevertheless, predictions of which protein (E1 or E2) acts as the HCV fusion protein remain controversial. Potential structural homology between the more C-terminally located E2 glycoprotein of HCV with other fusion proteins from the same family as well as with other type II fusion proteins suggests that E2 is a class II membrane fusion protein [54,55]. On the other hand, sequence analyses suggested that the ectodomain of E1 contains a fusion peptide-like motif, which spans residues 264 to 290, similar to the fusion peptides of paramyxoviruses and flaviviruses [56]. Proteomic computational analyses also suggested that E1 of HCV and E2 of pestiviruses are truncated class II fusion proteins [57].

It is now generally thought that the concerted actions of multiple membranotropic segments in fusion proteins are required for the fusion process [50,58,59]. Several regions in the ectodomain of E2, such as segments 430~449, 543~560, and 603~624 (or 603~634), were shown to possess a membrane-perturbing ability [60,61]. In assessing the peptide libraries of E1 and E2, Villalaín's group identified several membrane-active segments in E1 and E2, suggesting that both E1 and E2 participate in the fusion process [62]. Cosset's group recently identified six regions in E1 and E2 with features of fusion peptides and proposed that at least three regions, i.e., residues 270 to 284, which constitute the fusion peptide-like domain of E1, and residues 416~450 and 600~620 in E2 play a role in membrane fusion [63]. Nevertheless, Drummer's group mutated hydrophobic residues in this region and found that only the F285A mutant abolished viral entry whereas other mutations did not or only partially inhibit viral entry [64]. They thus suggested that E1 may not function in an analogous fashion to other class II fusion glycoproteins during membrane fusion. It is therefore important to characterize the involvement of other residues in this fusion in the viral entry process.

In the present study, we conducted mutagenesis coupled with a human immunodeficiency virus type 1 (HIV-1)/HCV E1E2 pseudotype approach and a cell-based fusion assay to address the involvement of the fusion peptide-like domain within E1 in early steps of virus-host cell interactions. We focused on those conserved residues among HCV isolates and shared by the E protein of flaviviruses or the fusion peptide of paramyxoviruses. We found that the severity of reduction in membrane fusion and viral entry by these mutations paralleled the conserved nature of these residues among HCV isolates. The results also showed that specific residues, but not the structure, of this fusion peptide-like motif are required for cell fusion and viral entry.

## Methods

### Cells and antibodies

Human embryonic kidney 293T and human hepatocarcinoma Huh7 cells were grown in Dulbecco's modified Eagle medium (DMEM) (Invitrogen; Carlsbad, CA) supplemented with 2 mM L-glutamine, 100 U/ml penicillin, 100 mg/ml streptomycin, and 10% fetal bovine serum (FBS). In particular, nonessential amino acids were also added to the Huh7 culture medium. Mouse monoclonal antibodies (MAbs) directed against HCV E1 (cat. # C65198M) and E2 (cat. # C65167M) were purchased from Biodesign (Saco, ME), and the rabbit anti-HCV E2 peptide antibody (cat. # GB10416) was from Genesis Biotech (Xindian, Taipei County, Taiwan). H53 MAb was an E2 conformation-specific MAb [6,65]. Hybridoma 183 (clone H12-5C) secretes an MAb that specifically recognizes HIV-1 capsid protein p24 as previously described [66]. Affinity-purified fluorescein isothiocyanate (FITC)-conjugated anti-mouse and anti-rabbit immunoglobulin G (IgG) was purchased from Kirkegaard & Perry Laboratories (Gaithersburg, MD).

### Construction of plasmids

To construct pcDNA3-E1E2, which expresses the E1 and E2 proteins of the H77 strain, a polymerase chain reaction (PCR) overlap extension method using pHCMV-E1HA and pHCMV-E2HA [67] as the templates was performed. The outer sense and antisense primers used were 5'-CCCGGTACCGCCGCCGCCATGAATTCCGACCTCATGGGGTAC-3' (which also encodes a Kozak sequence and 60 aa of the C-terminal core as the leader peptide for E1) and 5'-CCTCCAGATTAGCCCTCCGCTTGGGATATGAGTAACATCATCCA-3', respectively. The internal sense and antisense primers used were 5'-CTGCTATTTGCCGGCGTCGACGCGGAAACCCACGTCACCGGGGAAGT-3' and 5'-ACTTCCCCCGGTGACGTGGGTTTCCGCGTCGACGCCGGCAAATAGCAG-3', respectively. These primers encoded the junction sequence between E1 and E2. The KpnI- and XbaI-cut DNA fragment was cloned in the same sites in pcDNA3 (Invitrogen) to yield pcDNA3-E1E2. To construct the E1 fusion peptide-like domain mutants, the PCR overlap extension method using pcDNA3-E1E2 as the template was performed. Oligonucleotides 5'-CCATGTCACCAATGATTGCC-3' and 5'-CCTTCGCCCAGTTCCCCCACC-3' were used as the outer sense and antisense primers, respectively. Internal paired primers used to construct these mutants were: G267A, 5'-GATCTGCTTGTCGGGAGCGCCACCCTCTGC-3' (sense) and 5'-GCAGAGGGTGGCGCTCCCGACAAGCAGATC-3' (antisense); C272A, 5'-GGGAGCGCCACCCTCGCCTCAGCCCTCTACGTG-3' (sense) and 5'-CACGTAGAGGGCTGAGGCGAGGGTGGCGCTCCC-3' (antisense); G278A, 5'-TCAGCCCTCTACGTGGCCGACCTGTGCGGGTCT-3' (sense) and 5'-AGACCCGCACAGGTCGGCCACGTAGAGGGCTGA-3' (antisense); D279N, 5'-CTCTACGTGGGGAACCTGTGCGGGTCTGTTTTT-3' (sense) and 5'-AGACCCGCACAGGTTCCCCACGTAGAGGGCTGA-3' (antisense); C281A, 5'-TACGTGGGGGACCTGGCCGGGTCTGTTTTTCTT-3' (sense) and 5'-AAGAAAAACAGAGGCGCACAGGTCAAACACGTA-3' (antisense); G282A, 5'-GGGGACCTGTGCGCCTCTGTTTTTCTTGTTGGT-3' (sense) and 5'-AAGAAAAACAGAGGCGCACAGGTCCCCCACGTA-3' (antisense); and G288A, 5'-GTTTTTCTTGTTGCCCAACTGTTTACCTTCTCT-3' (sense) and 5'-GGTAAACAGTTGGGCAACAAGAAAAACAGACCC-3' (antisense). The PCR fragments were digested with XhoI and BamHI, and then used to replace the homologous sequence in WT pcDNA3-E1E2 to generate each of the mutant plasmids.

To construct pCAGGS-sCD81/LEL-Ig, which encoded the large extracellular loop (LEL) of human CD81 (residues 123~201) flanked by the 25-aa-leader peptide of alkaline phosphatase and the CH_2 _and CH_3 _domains of the human IgG Fc receptor at the N- and C-termini, respectively, a series of 5' sense overlapping primers of 5'-TTCTAGACCACCATGCTGTTACTCTTGCTGTTACTGGG-3', 5'-TTGCTGTTACTGGGCCTGAGGCTGCAGCTGTCCTTAGGT-3', 5'-CAGCTGTCCTTAGGTATCATCAGAACACGAGCAAAACGA-3', and 5'-ACACGAGCAAAACGATTTGTCAACAAGGACCAGATCG-3', and the 3' antisense primer 5'-TGAATTCTTCCCGGAGAAGAGGTCATCG-3' were used to amplify the coding sequences indicated above in an assembled PCR using pcDNA3.1-hCD81-V5-His-Topo [68] as the template. The XbaI- and EcoRI-digested DNA fragment was used to replace the coding sequence of angiotensin-converting enzyme 2 cloned in pCAGGS-ACE2-CH_2_CH_3_-Ig [69] to generate pCAGGS-sCD81/LEL-Ig.

### Plasmid DNA transfection

For expression of HCV E1E2, 3 × 10^6 ^293T cells were seeded into 10-cm Petri dishes and cultured for 24 h prior to transfection. Subconfluent 293T monolayers were transfected with 10 μg of wild-type (WT) or mutant E1E2-expressing plasmids by a standard calcium phosphate coprecipitation method. For the pseudotyping analyses, 293T cells were cotransfected with 10 μg each of an *env*-defective, pNL4-3R^-^E^-^Luc reporter provirus [70], in which a firefly luciferase gene replaces the *nef *gene, and WT or mutant E1E2 plasmids using the calcium phosphate coprecipitation method (for analysis of viral protein expression) or Lipofectamine 2000 (Invitrogen) according to the manufacturer's procedures (for generating pseudovirions).

### Fluorescence-activated cell sorter (FACS) analysis

To assess E1E2 expression, two sets of 293T cells (10^6^/dish) seeded in 6-cm Petri dishes were cultured for 24 h and then transfected with 10 μg of WT or mutant E1E2 plasmids by the calcium phosphate coprecipitation method. Two days after transfection, cells were detached with phosphate-buffered saline (PBS) containing 0.05% EDTA and washed twice with PBS containing 0.2% FBS. Cells from one set were used to determine total E1E2 expression, and those from the other set were processed for E1E2 cell-surface expression. For total protein expression, cells were fixed in 4% paraformaldehyde at 4°C for 30 min and permeabilized with 0.25% Triton X-100 at 4°C for 5 min. Cells were subsequently incubated with 100 μl of E1 MAb at a concentration of 10 μg/ml at 4°C for 2 h. After three washes, cells were incubated with 100 μl of FITC-conjugated anti-mouse IgG at a 1:100 concentration in PBS containing 0.2% FBS at 4°C for 1 h. Immunostained cells were washed with PBS containing 0.2% FBS, resuspended in 500 μl of PBS, and quantitated by a FACSCaliber (Becton Dickinson Immunocytometry Systems; San Jose, CA) using the Flow Jo software (Tree Star, Inc., Ashland, OR). For cell surface expression, cells were successively incubated with 10 μg/ml of the E1 MAb and FITC-conjugated anti-mouse IgG and fixed with 4% paraformaldehyde; stained cells were then analyzed by flow cytometry. Alternatively, transfected cells were examined for total and cell surface expression of E2 using a rabbit anti-E2 antibody and appropriate secondary antibody.

### Soluble CD81 (sCD81) pull-down assay

Sub-confluent 293T cells grown in 10-cm Petri dishes were transfected with 10 μg of pCAGGS-sCD81/LEL-Ig, and cell-free culture supernatants were concentrated 100-fold by centrifugation through Centricon 70 membranes (Millipore; Billerica, MA) at 1000 × *g *at 4°C and used as the source of sCD81. At the same time, 293T cells grown in 10-cm dishes were transfected with 20 μg of pcDNA3, WT, or mutant E1E2 plasmids. Two days after transfection, cells were lysed with cold PBS containing 1% CHAPSO. Cell lysates were incubated with or without 100 μl of concentrated sCD81/LEL-Ig followed by incubation with 50 μl of protein A-Sepharose 4B beads at 4°C for 2 h. After washing twice with PBS containing 0.5% CHAPSO, the co-precipitated proteins were eluted from protein A-Sepharose beads with 50 μl of 2× Laemmli sample buffer by heating to 95°C for 5 min, and the eluted proteins were resolved by 10% sodium dodecylsulfate (SDS) polyacrylamide gel electrophoresis (PAGE) followed by Western blotting with E1 and E2 MAbs, respectively.

### SDS-PAGE and Western immunoblotting analysis

To determine E1E2 expression, 2 days after transfection, cells were lysed with cold lysis buffer [PBS containing 1% each of Nonidet P-40 and sodium deoxycholate and the protease inhibitor cocktail (Roche, Basel, Switzerland)]. After centrifugation at 18,000 × *g *at 4°C for 10 min to remove cell debris, the supernatants were subjected to 10% SDS-PAGE followed by Western blotting using E1- and E2-specific MAbs, respectively. For incorporation of HCV E1E2 into HIV-1 virions, 2 days after transfection, viruses were isolated from culture supernatants by ultracentrifugation at 160,000 × *g *for 90 min at 4°C through 2 ml of a 20% sucrose cushion in a Beckman/Coulter (Fullerton, CA) SW41 rotor as previously described [71]. Equal volumes of cell and virion lysates were subjected to reducing SDS-PAGE followed by immunoblot analysis using HCV E1 and E2 MAbs, and HIV-1 capsid p24 MAb 183, respectively.

### Metabolic labeling and immunoprecipitation

Two days after transfection, 293T cells expressing WT or mutant E1E2 proteins were metabolically labeled with [^35^S]methionine (NEN Life Science Products; Boston, MA) for 30 min and chased with excess cold methionine as described previously [72] for 4 h. At the indicated chase times, cells were lysed with PBS containing 1% CHAPSO and the complete protease inhibitor mixture. After standing at 4°C for 10 min, the cell lysates were cleared by centrifugation at 10,000 × *g *at 4°C for 10 min. Equal volumes of cell lysates were precipitated with 10 μl of anti-E2 MAb H53. The antibody was prebound to a mixture containing 30 μl of protein A-Sepharose 4B (Amersham Biosciences). After incubation at 4°C for 2 h, the immune complexes were washed six times with RIPA buffer (10 mM Tris-HCl, pH 7.2, containing 1% Triton X-100, 0.1% SDS, 1% sodium deoxycholate, 5 mM EDTA, and 0.15 M NaCl) and then boiled in 30μl of Laemmli buffer. The immunoprecipitates were examined by nonreducing 10% SDS-PAGE.

### Production of HCVpp and viral entry assay

Culture supernatants were collected from 293T cells cotransfected with pNL4-3R^-^E^-^Luc and HCV WT or mutant E1E2 expression plasmids at 24, 48, and 72 h after transfection, combined, filtered through 0.45-μm membrane discs, and concentrated by centrifugation at 100,000 × *g *for 2 h at 4°C in an SW28 rotor. Viral pellets were resuspended in DMEM containing 2.5% FBS to concentrate the viral particles by 100-fold. Pseudovirions were normalized by reverse transcriptase (RT) activity as previously described [73]. Huh7 cells seeded in 48-well plates (2 × 10^4^/well) were cultured for 24 h, and then infected with the WT or mutant pseudotypes containing 5 × 10^5 ^cpm of RT activity in the presence of 8 μg/ml polybrene at 37°C overnight. Each infection assay was performed in triplicate wells. After removing the viral suspensions, cells were incubated at 37°C for an additional 48 h before being lysed with 60 μl per well luciferase cell lysis buffer (Promega, Madison, WI). Fifty microliters of lysates was added to 50 μl of luciferase substrate solution (Promega), and the luciferase activity, in terms of relative light units, was measured for 10 s in a Sirius luminometer (Berthold Detection Systems, Pforzheim, Germany).

### Cell-to-cell fusion assay

293T (5 × 10^5^/well) and Huh7 (2 × 10^5^/well) cells were seeded in 6-well plates 1 day prior to the assay. As effector cells, 293T cells were cotransfected with 2 μg of pCAG-T7pol [74], a plasmid carrying the T7 RNA polymerase gene under the control of the CAG promoter, and 1 μg each of the WT and mutant E1E2 plasmids. For target cells, Huh7 cells were transfected with 2 μg of pT7EMCVLuc [74], which encodes the firefly luciferase gene under control of the T7 promoter. Forty-eight hours after transfection, 293T effector cells were detached from the dishes by treatment with 0.05% EDTA in PBS and resuspended in DMEM containing 10% FBS. 293T effector cells were overlaid onto the target cells, and the cultures were incubated for 5 h. The cocultures were bathed in PBS at pH 5.0 for 2 min at 37°C, and then incubated with DMEM containing 10% FBS for an additional 5 h. Each cell fusion assay was performed in triplicate wells. Cells were harvested, lysed, and assayed for firefly luciferase activity.

### Pseudotype binding assay

Binding of HCVpp to Huh7 cells was performed as previously reported [63]. Huh7 cells (3 × 10^5^/well) seeded in 12-well plates were cultured for 24 h before HCVpp binding. Cells were incubated with WT or mutant HCV pseudotypes containing 10^7 ^cpm of RT activity in a total volume of 300 μl of DMEM containing 10% FBS and 0.1% of NaN_3 _at 37°C for 1 h. After three washes with 1 ml of PBS containing 2% FBS and 0.1% NaN_3_, cells were incubated with 4 μg of rabbit anti-E2 in a total volume of 300 μl on ice for 2 h. After washing, cells were incubated with a 1:100 concentration of FITC-conjugated anti-rabbit IgG on ice for 1 h. After washing, cells were fixed with 300 μl of 4% paraformaldehyde on ice for 30 min, and then analyzed by FACS. Alternatively, cells were fixed, permeabilized, and then stained with rabbit anti-E2 or anti-p24. After incubation with FITC-conjugated anti-rabbit IgG, the immunostained cells were analyzed by FACS.

## Results

### Construction and expression of E1 fusion peptide-like motifmutants

**Figure 1:**
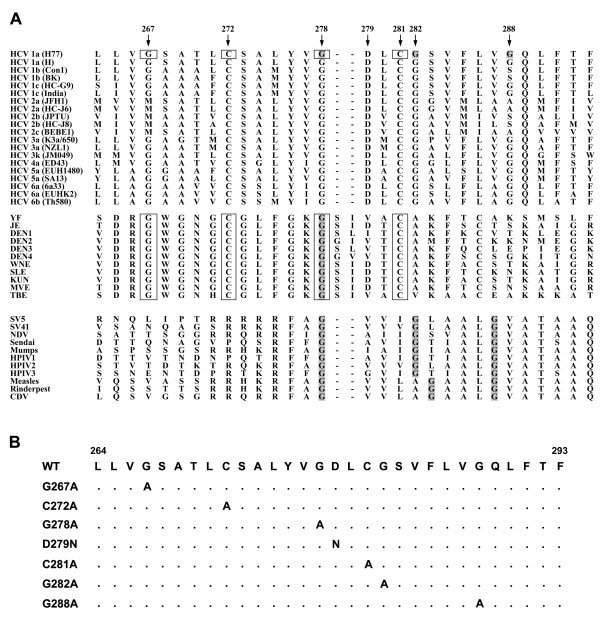
**Analysis of the HCV E1 putative fusion domain**. (A) Alignment of the E1 fusion peptide-like motif of HCV with the predicted fusion peptide sequences of the E protein of flaviviruses and fusion protein of paramyxoviruses. The E1 putative fusion peptide sequence derived from various HCV genotypes contains similarities to the predicted fusion peptide of flavivirus E glycoproteins which are boxed. The spacing of Gly residues, as indicated by shading in the HCV putative fusion peptide, is also similar to those within the fusion peptides of paramyxovirus F proteins. (B) Construction of E1 fusion peptide-like domain mutants. Construction of E1 mutants with substitutions of Ala or Asn for the residues as indicated in the fusion peptide-like domain was performed by a site-specific, oligonucleotide-directed PCR overlap extension method as described in "Materials and Methods". Dots in the sequence indicate that the amino acid in that position is identical to that of the WT sequence.

To understand the involvement of the E1 putative fusion domain, which spans residues 264~290, in viral infection, we characterized the effects of amino acid substitutions for conserved residues in this region on viral infection. This internal hydrophobic region of E1 bears similarities to the predicted fusion peptide from E proteins of flaviviruses [56]. First, two Cys residues located at positions 272 and 281 are highly conserved among HCV genotypes and all flavivirus E protein sequences analyzed (Fig. 1, top and middle panels), suggesting their role in disulfide bond formation. Second, the Gly residue at 278 in E1 is highly conserved among all genotypes, whereas Gly-267 exists in all but genotype 2, and in the predicted fusion peptides of all flavivirus E proteins examined (Fig. 1, top and middle panels). Third, an Asp residue at position 279 is present in all HCV genotypes and some of the flavivirus E sequences (Fig. 1, top and middle panels). The presence of acidic residues in the fusion peptides was also noted in some low-pH-activated viral fusion proteins [75]. In addition, alignment of the E1 fusion peptide-like domain with the fusion peptide of F proteins from paramyxoviruses also reveals that the Gly residues between these two classes of fusion domains are similar, i.e., Gly-278 and Gly-282 in HCV are highly conserved with those derived from paramyxoviruses, while Gly-288, which is found in some HCV genotypes, is also found in the fusion protein of paramyxoviruses (Fig. 1A, first and third panels).

Accordingly, substitutions were introduced at these conserved residues, and their effects on the phenotypes of E1E2 proteins were examined. In the first group, mutants were constructed by Ala or Asn substitutions for the conserved residues located in the N-terminal half of this region, i.e., G267A, C272A, G278A, D279N, and C281A. The first single-letter amino acid code indicates that the residue located at that position was replaced by an Ala (A) or Asn (N) residue, as shown by the second code letter. Another group contained Ala substitutions for the conserved Gly residues located in the C-terminal segment of this region, i.e., G278A, G282A, and G288A mutants. All of these mutants were constructed by oligonucleotide-directed PCR overlap extension mutagenesis (Fig. 1B) from a pcDNA3-based E1E2 expression plasmid derived from a genotype of the 1a H77 strain [67].

**Figure 2:**
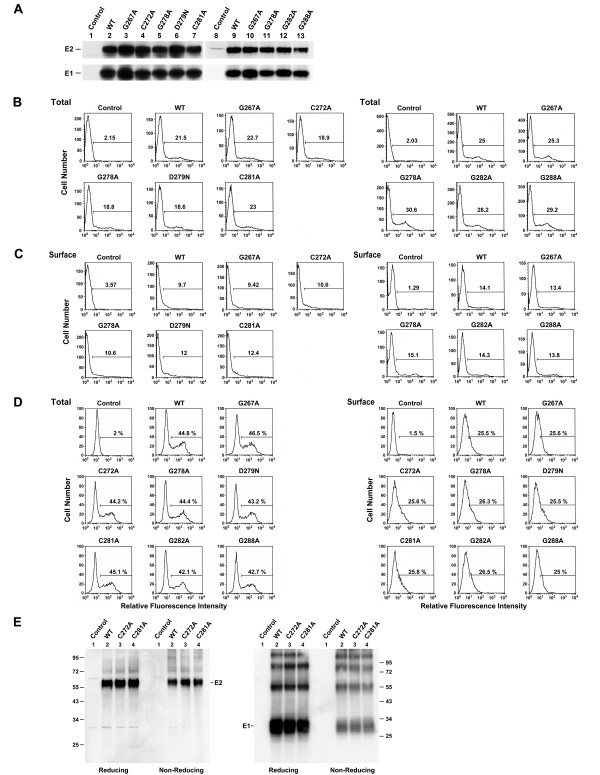
**Expression of E1 mutants**. (A) Western blotting analysis. 293T cells were transfected with pcDNA3, marked as the control, or with each of the pcDNA3-dervied WT and mutant E1E2 plasmids. Cell lysates containing equal total proteins prepared 2 days after transfection were subjected to reducing SDS-PAGE followed by Western blotting using E2- and E1-specific MAbs, respectively. (B to D) Analyses of mutant protein expression by FACS. 293T cells in two sets were transfected with pcDNA3 (marked as the control) or with each of the WT and mutant proteins. Two days after transfection, one set of cells was assessed for total E1 expression (B) and the other for E1 cell surface expression (C) using an E1 MAb. In another separate experiment, transfected cells were assessed for the total and cell surface expressions of E2 with a rabbit anti-E2 antibody (D). (E) Analyses of Cys-substituted mutant proteins by reducing and nonreducing SDS-PAGE. 293T cells transfected with pcDNA3 (marked as the control), WT, C272A, and C281A mutant plasmids were resolved by reducing or nonreducing conditions as indicated. The Western blots were then analyzed with E1- and E2-specific MAbs, respectively. The sizes in kDaltons of standard proteins were indicated to the right.

To understand whether these E1 mutant proteins are normally expressed and processed, 293T cells were transfected with each of the WT and mutant plasmids, and cell lysates were subjected to SDS-PAGE followed by Western blotting using MAbs specific for E1 and E2. All N-terminal half mutants, i.e., G267A, C272A, G278A, D279N, and C281A, produced comparable levels of E1 and E2 proteins to those produced by the WT plasmid upon transfection (Fig. 2A, compare lanes 3~7 to lane 2). Similarly, a steady-state expression analysis also showed that similar amounts of E1 and E2 to those produced by the WT and G267A mutant were detected for the three C-terminal half mutants, i.e., G278A, G282A, and G288A mutants (Fig. 2A, compare lanes 11~13 to lanes 9 and 10).

To confirm these results, 293T cells expressing WT or mutant proteins were determined for the total E1 protein by FACS. Mutations in the N-terminal half of this region did not greatly affect E1 expression (Fig. 2B, left panel). Likewise, similar amounts of E1 were also detected for C-terminal half mutants compared to that produced by the WT plasmid upon transfection (Fig. 2B, right panel).

We then determined whether these mutant proteins were effectively expressed on the cell surface by flow cytometry using an anti-E1 MAb. Similar levels of E1 protein of N-terminal half mutants to that of the WT protein were expressed on the cell surface (Fig. 2C, left panel). C-terminal half mutants also produced comparable levels of E1 on the cell surface, as opposed to the WT protein (Fig. 2C, right panel).

Next, the total and cell surface expressions of E2 expressed by these mutants was examined along with the WT protein using a rabbit anti-E2 antibody. Mutations in this region did not affect the levels of intracellular or cell surface-bound E2 (Fig. 2D, left and right panels, respectively). The low levels of E1 and E2 detected on the cell surface were not unexpected since HCV E1 and E2 are predominantly retained in ER after synthesis and only small amounts of E1 and E2 escape ER retention and are transported to the cell surface [15,18,76]. These results together indicate that mutations in this region did not greatly affect the synthesis of or processing into E1 and E2 proteins, nor did they alter the cell surface expression of E1 and E2.

### Characterization of the disulfide bond formation of Cys-272 and Cys-281

To examine whether mutations at Cys-272 and Cys-281 may be involved in intra- or extramolecular disulfide bond formation, cell lysates expressing WT or each of the mutant proteins were subjected to reducing and non-reducing SDS-PAGE followed by Western blotting using E2 and E1 MAbs, respectively. As shown in Fig. 2E, substitutions of Ala for Cys-272 and Cys-281, respectively, did not affect the mobility of E1 or E2 under reducing or non-reducing SDS-PAGE (Fig. 2E). Moreover, these two mutations did not alter the migration profiles of E1 or E2, compared to those of the WT protein (Fig. 2E). These observations rule out the possibility that these two Cys residues in E1 are involved in disulfide bond formation.

### The cell-cell fusion ability of mutant proteins

**Figure 3:**
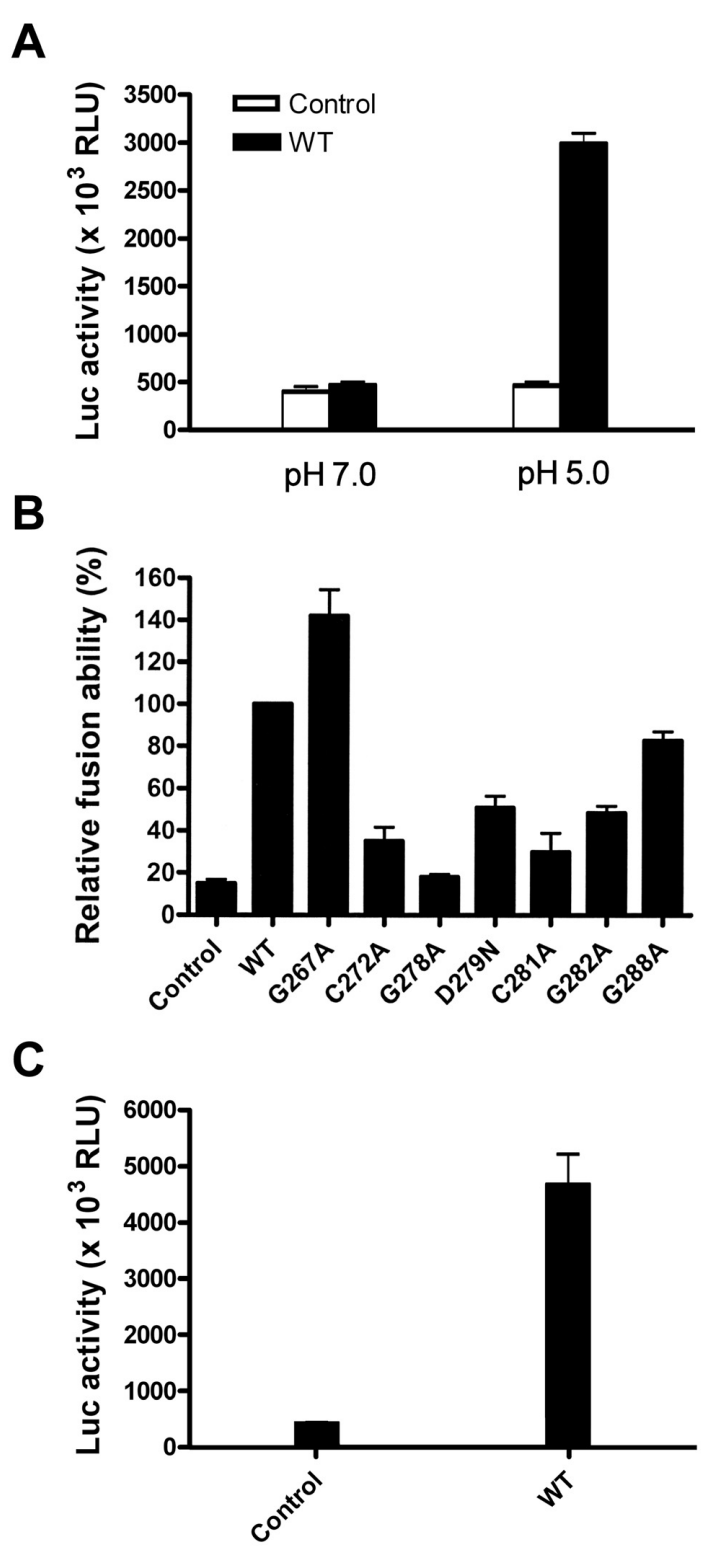
**Membrane fusion ability of mutant proteins**. (A) Fusion ability of WT E1 and E2 at neutral and acidic pHs. 293T cells were cotransfected with pCAG-T7Pol and pcDNA3 (marked as the control) or the WT E1E2 plasmid as indicated, and Huh 7 cells were transfected with pT7EMCVLuc. Two days post-transfection, cells were detached, resuspended, and added to transfected Huh 7 cells. Five h after coculture, cells were treated with PBS at pH 7.0 and pH 5.0, respectively, and 37°C for 2 min, and then incubated for an additional 5 h. Cells were lysed and assayed for firefly luciferase activity. The mean values of luciferase activities from three separate analyses with standard deviation are shown. (B and C) The membrane fusion ability of mutant proteins. 293T cells were cotransfected with pCAG-T7Pol and pcDNA3 (marked as the control), WT, or mutant E1E2 plasmids, and the membrane fusion abilities of WT and mutant proteins were assessed at pH 5.0 as described in (A). The luciferase activity was measured, and the relative fusion abilities of the mutants were expressed as a percentage of that of the WT E1E2 (B). The actual values of luciferase activities of the control and WT samples shown in (B) are depicted in (C). All the diagrams represent the results from three independent experiments (mean ± standard deviation), each of which was performed with triplicate samples.

Since the E1E2 proteins of all mutants were expressed on the cell surface as effectively as were the WT proteins, we then determined whether mutations in the E1 fusion peptide-like domain affected the cell fusion ability of E1E2 by a cell-based T7 polymerase/T7 promoter-driven luciferase reporter gene activation assay as previously reported [74,77]. As a control, a bath of cocultured effector and target cells at pH 7.0 did not mediate cell fusion; however, exposure of cocultured cells to pH 5.0 induced membrane fusion, as shown by detection of luciferase activity (Fig. 3A). In addition, 293T cells transfected with the WT E1E2 plasmid without pCAG-T7pol did not show cell fusion either even cocultured cells were bathed at pH 5.0 (data not shown). The G267A mutant showed a higher level of cell fusion than did the WT protein (Fig. 3B). However, the cell fusion abilities of the other N-terminal half mutants were reduced to a level ranging from 18% (the G278A mutant) to 51% (the D279N mutant) of that of the WT protein, whereas the control without E1E2 expression showed a background level of 15% of that of the WT E1E2 (Fig. 3B). For C-terminal half mutants, Ala substitution for Gly-288 did not greatly affect the fusion ability, while Ala substitution for Gly-282 decreased the fusion ability to 48% of that of the WT E1E2 (Fig. 3B). The actual values of luciferase activities shown in the control and WT samples from three separate analyses are also depicted in Fig. 3C.

### Assessment of binding of mutant proteins to sCD81

**Figure 4:**
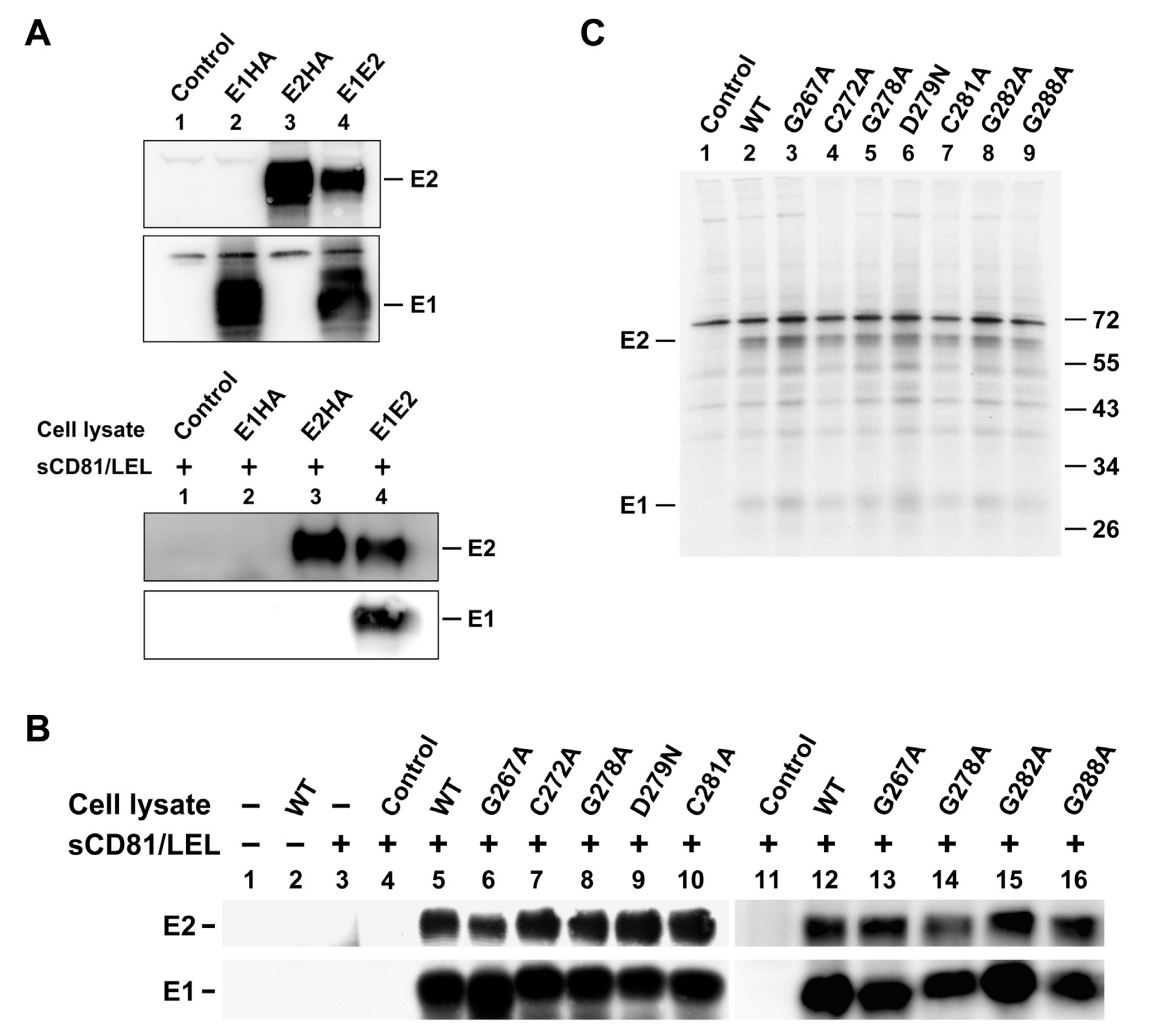
**Analysis of the CD81-binding ability of E2 and of the E1-E2 interactions in mutant proteins**. (A) 293T cells were transfected with pcDNA3 (marked as the control), pHCMV-E1HA, pHCMV-E2HA, and WT pcDNA3-E1E2 plasmids, respectively. Two days after transfection, cells were lysed with buffer containing 1% CHAPSO. A portion of the cell lysates was directly resolved by reducing SDS-PAGE followed by Western blotting using E1 and E2 MAbs, respectively (top panel). Another portion of the lysates was incubated with equal volumes of concentrated sCD81-LEL followed by incubation with protein A-Sepharose beads. The precipitated proteins were resolved by reducing SDS-PAGE followed by Western blotting using E2 and E1 MAbs, respectively (bottom panel). (B) 293T cells were transfected with pcDNA3 (marked as the control) or with each of the WT and mutant E1E2 plasmids. Two days after transfection, cell lysates were incubated with or without concentrated sCD81-LEL followed by incubation with protein A-Sepharose beads. The isolated proteins were resolved by reducing SDS-PAGE followed by Western blotting using E2 and E1 MAbs, respectively. (C) Coprecipitation of E1 and E2 with a conformation-dependent E2 MAb H53. 293T cells were transfected with pcDNA3 (marked as the control), WT, or mutant E1E2 plasmids. Transfected cells were metabolically labeled with [^35^S]methionine for 30 min and chased for 4 h. Cell lysates were successively incubated with the H53 MAb and protein-A-Sepharose beads, and the isolated proteins were subjected to nonreducing SDS-PAGE followed by fluorography.

To study whether the reduced membrane fusion ability of mutants was due to reduced E2-CD81 and/or E1-E2's interaction with mutants, we first constructed a plasmid encoding the LEL of CD81 flanked by the 25-aa leader peptide of the secreted alkaline phosphatase and the human immune IgG Fc receptor at its N and C termini, respectively. This plasmid was transfected into 293T cells to produce secreted sCD81/LEL. Next, cell lysates containing WT or mutant proteins were incubated with equal amounts of concentrated sCD81/LEL followed by incubation with protein A-Sepharose beads. The precipitated proteins were analyzed by Western blotting using E1 and E2 MAbs, respectively. To assess the specificity of this assay in detecting coprecipitation of E1 by sCD81/LEL through E1-E2 interactions, E1HA, E2HA, and E1E2 were separately expressed (Fig. 4A, top panel) and then coprecipitated with sCD81/LEL (Fig. 4A, bottom panel). E1 was bound to sCD81/LEL only when coexpressed with E2 (Fig. 4A, bottom panel, lane 4). Also, the WT E1 and E2 proteins coprecipitated with protein-A Sepharose beads only when sCD81/LEL had been added to cell lysates (Fig. 4B, lane 5). Similar amounts of E1 and E2 proteins of N-terminal half mutants to those of the WT proteins were bound to sCD81/LEL (Fig. 4B, compare lanes 6~10 to lane 5). In addition, mutations in the C-terminal half of the fusion peptide-like motif did not greatly affect the coprecipitation of E1 and E2 with sCD81/LEL (Fig. 4B, compare lanes 13~16 to lane 12). These results together indicate that mutations in this region did not affect binding of E2 to CD81 or the E1-E2 association.

### Formation of non-covalent E1-E2 heterodimeric complexes

To understand whether mutant E1 can form a non-covalent heterodimer with E2, lysates of [^35^S]methionine-labeled cells expressing envelope glycoproteins were precipitated with H53, an E2 conformation-dependent MAb. The proteins isolated were resolved by nonreducing SDS-PAGE. Similar levels of E2 were detected from WT and mutant plasmid transfections (Fig. 4C), indicating that mutations in the fusion peptide-like domain of E1 do not affect the folding and assembly of E2. Also, comparable levels of E1 were coprecipitated with E2 from WT and mutant protein expressions (Fig. 4C), indicating that these mutations neither affect the formation of a non-covalent hetero-dimer with E2 nor alter the folding and assembly of E1.

### Viral entry properties of pseudotypes bearing E1 mutants

**Figure 5:**
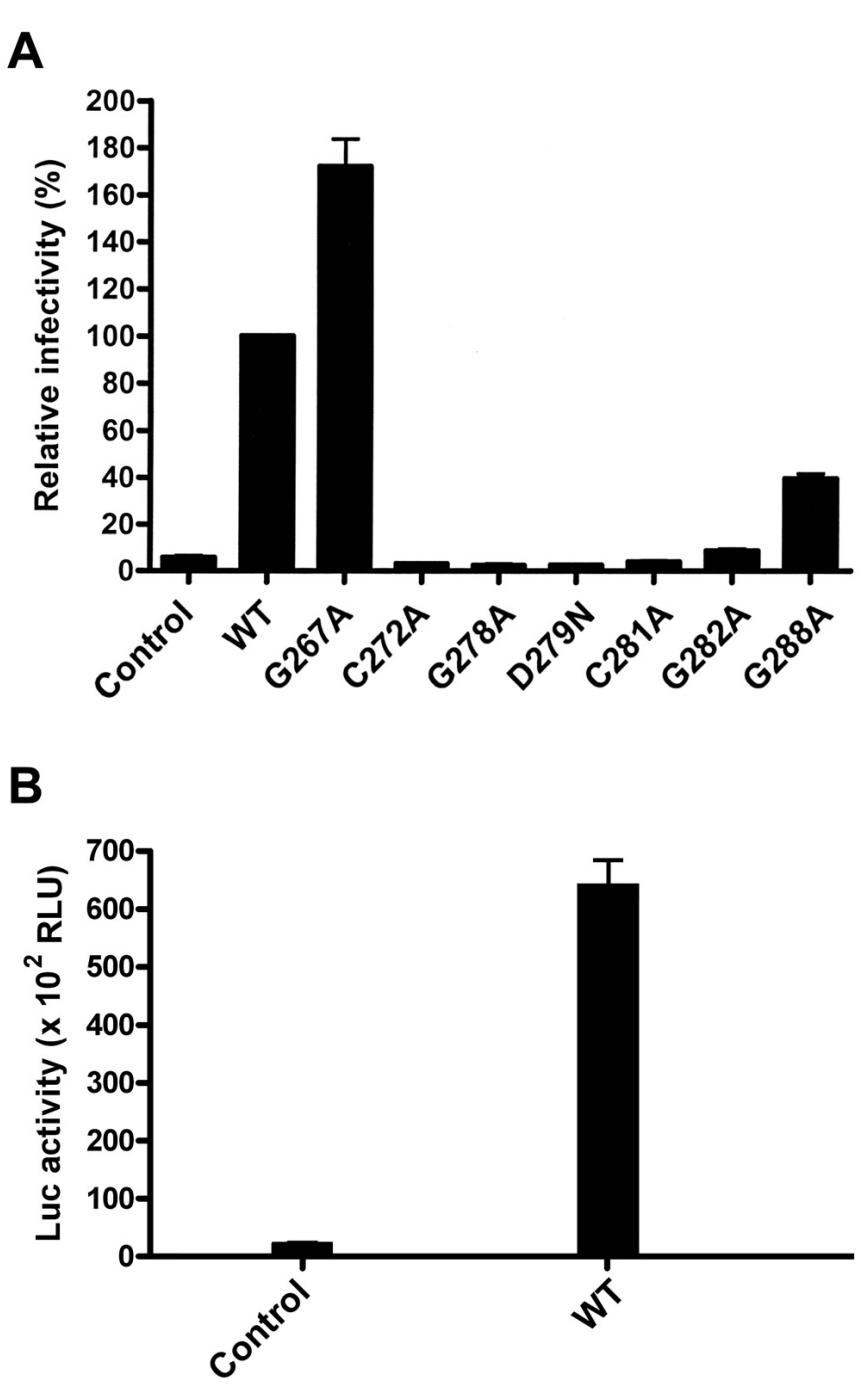
**Assessment of the viral entry ability of HCV pseudotypes**. Cell-free, *env*-defective NL4-3R^-^E^-^Luc reporter viruses produced from cotransfection with pcDNA3 (marked as the control) or with WT or mutant E1E2 plasmids were normalized for RT activity prior to challenge with Huh7 cells. Two days after infection, cells were assayed for luciferase activity, and the relative viral entry ability mediated by mutant proteins was expressed as a percentage of that mediated by WT E1E2. Results from three independent experiments are shown as the mean ± standard deviation (A). The means of the actual luciferase activities of the control and WT samples with standard deviations are also shown in (B).

To study whether E1 fusion peptide-like domain mutants are able to mediate viral entry into host cells, the ability of these mutants to support an *env*-deficient, luciferase gene-encoding HIV-1 reporter virus was examined. 293T cells were cotransfected with pNL4-3R^-^E^-^Luc and each of the WT or mutant E1E2 plasmids. Cell-free pseudotypes were normalized by RT activity prior to challenge with Huh7 cells, and luciferase activity was measured 2 days after infection. Except for the G267A mutant, which showed an increased viral entry ability compared to WT E1E2, the one-cycle virus infectivity of all N-terminal half mutants was abrogated to below the background level, i.e., 6% of that mediated by WT E1E2 (Fig. 5A). When the viral entry ability of C-terminal half mutants was examined, the G278A mutant also strikingly reduced its entry ability to below the background level, while the G282A and G288A mutants showed reduced *trans*-complementation abilities to 9% and 40%, respectively, of that mediated by WT E1E2 (Fig. 5A). The actual luciferase activities of pseudotypes generation from pcDNA3 or WT E1E2 plasmid cotransfection are shown in Fig. 5B.

### Incorporation of mutant E1E2 into HIV-1-like particles

**Figure 6:**
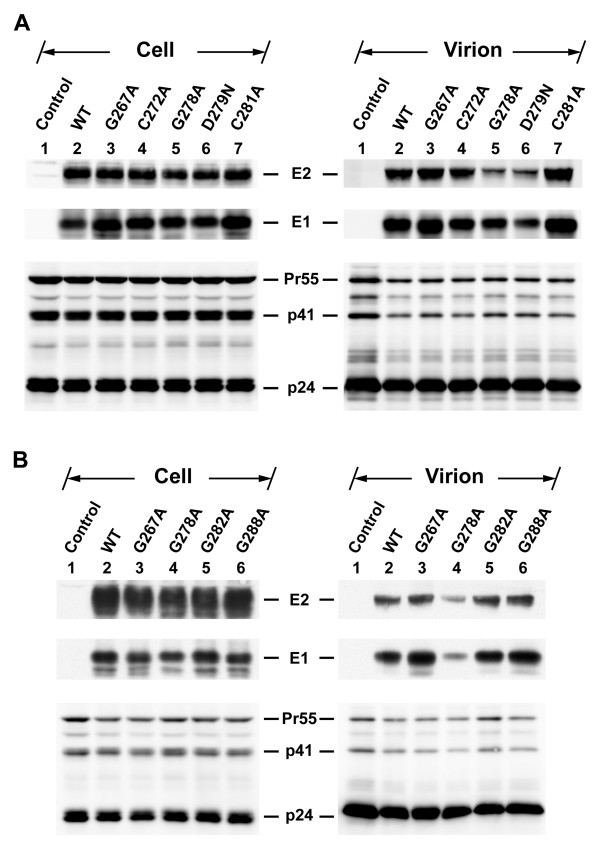
**Incorporation of WT and mutant E proteins into HIV-1-like particles**. 293T cells were cotransfected with pNL4-3R^-^E^-^Luc and pcDNA3 (marked as the control) or with each of the WT and mutant E1E2 plasmids. Virions were isolated by sedimenting culture supernatants through a cushion containing 20% sucrose. Equal volumes of cell and virion lysates were analyzed by SDS-PAGE under reducing conditions followed by Western blotting using MAbs specific for E2, E1, and HIV-1 capsid p24, respectively.

To understand the nature of reduced viral infectivity of these mutant pseudotypes, cell and virion lysates obtained from cells cotransfected with pNL4-3R^-^E^-^Luc and each of the WT and mutant plasmids were analyzed by Western blotting. In the analysis of N-terminal half mutants, similar amounts of the Gag Pr55 precursor and its cleaved products, p41 and p25/p24, were detected in cells and virions of WT and mutant plasmid transfections (Fig. 6A). In addition, similar amounts of intracellular E1 and E2 proteins were detected for all mutants compared to those of WT proteins (Fig. 6A, left panel). With the exception of the G278A and D279N mutants, which showed reduced assembly of E1 and E2 into HIV-1-like particles compared to the WT E1E2 (Fig. 6A, right panel, compare lanes 5 and 6 to lane 2), the three G267A, C272A, and C281A mutants showed comparable amounts of E1 and E2 incorporation into the virus (Fig. 6A, right panel, compare lanes 3, 4, and 7 to lane 2). It is likely that Gly-278 and Asp-279 may have a role in maintaining an intact structure and/or in assembly of the E1E2 complex into HCVpp; therefore, mutations at these two residues resulted in decreased E1 and E2 assembly into the virus. When C-terminal half mutants were analyzed, Gag synthesis, precursor processing, and assembly/budding into virions were normal compared to WT E1E2 transfection (Fig. 6B). E1 and E2 proteins from all mutants were also expressed normally in cells (Fig. 6B, left panel). The G278A mutant again showed reduced incorporation of E1 and E2 into the HIV-1 pseudovirions (Fig. 6B, right panel, compare lane 4 to lane 2). The E1 and E2 proteins of G282A and G288A mutants were incorporated into pseudoparticles as effectively as were WT E1 and E2 proteins (Fig. 6B, right panel, compare lanes 5 and 6 to lane 2).

### Assessment of binding of HCVpp bearing respective mutant E1E2 proteins to Huh7 cells

**Figure 7:**
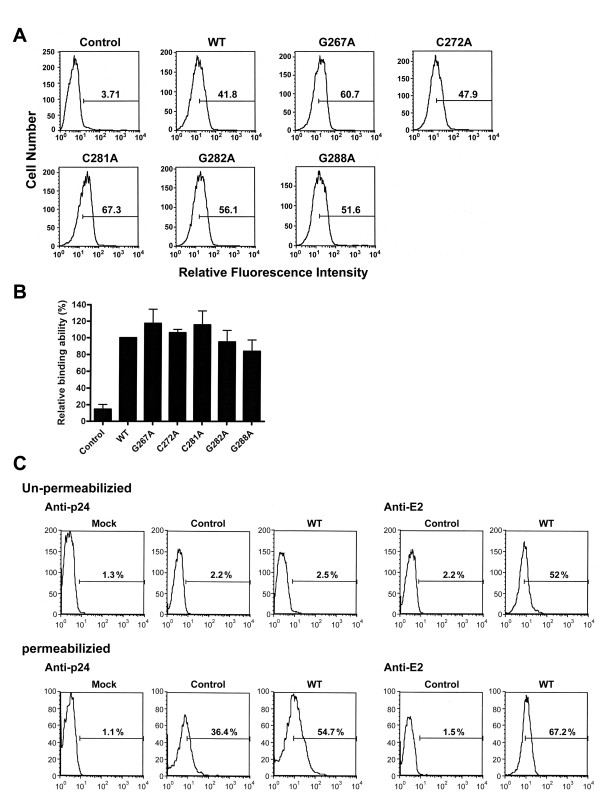
**Target cell-binding ability of pseudotypes bearing mutant proteins**. (A and B) Cell-free NL4-3R^-^E^-^Luc reporter viruses were produced from cotransfection with pcDNA3 (marked as the control) or with each of WT and mutant E1E2 plasmids, and normalized by RT activity prior to incubation with Huh7 cells. One h post-incubation, cells were successively incubated with a rabbit anti-E2 antibody and FITC-conjugated anti-rabbit IgG, fixed with paraformaldehyde, and then analyzed by flow cytometry (A). The relative binding ability of mutant pseudotypes is expressed as a percentage of that of the WT pseudotype. Results were quantified from three individual experiments with the standard deviation shown (B). (C) Huh7 cells were incubated with medium, HIV-1 particles without E1E2 (marked as the control), and the WT pseudotype, respectively. Cells were then divided into two groups; one group of cells was separately immunostained with a rabbit anti-HIV-1 p24 or rabbit anti-E2 and fixed, and immunostained cells were analyzed by FACS (top panel). Another group of cells was fixed, permeabilized with paraformaldehyde, and immunostained with rabbit anti-p24 and anti-E2, respectively, followed by FACS analysis (bottom panel).

To understand whether the reduced viral entry capacity of mutant pseudotypes was due to their insufficient binding to cell receptors, binding of HCVpp with alternative mutant E1E2 proteins to target Huh7 cells [63] was performed in the absence of permeabilization. Because G278A and D279N mutants exhibited reduced E1 and E2 assembly into HCVpp virions, they were excluded from the binding analysis. The WT and all mutant pseudotypes analyzed bound to Huh7 cells at comparable levels, as shown by flow cytometric analysis of bound pseudovirions in a representative experiment (Fig. 7A) or from three independent analyses (Fig. 7B). This binding measured E1E2-dependent binding to Huh7 cells since NL4-3R^-^E^-^Luc reporter virus devoid of E1E2 not bind to Huh7 cells (Fig. 7A and 7B).

It was reported that murine leukemia virus vector particles can bind to cells in the absence of Env-receptor interactions in an early step of virus attachment to cells [78]. We then examined whether the binding of WT HCVpp to Huh7 cells can occur without the specific interaction between E1E2 and HCV receptors. When the cell and virus culture was left unpermeabilized, the WT pseudotype was still bound to Huh7 cells, as evidenced by anti-E2 staining (Fig. 7C, compare profiles 4 and 5 in the top panel). As expected, no Gag signals were detected under this unpermeabilized condition (Fig. 7C, profiles 1 to 3 in the top panel). When the cell and virus culture was permeabilized, binding with the WT pseudotype showed a higher level of Gag signal than binding with the control virus without E1E2; the latter only showed a moderate Gag signal (Fig. 7C, compare profiles 2 and 3 in the bottom panel). Interestingly, a higher level of the E2 signal was detected under the permeabilized condition compared to binding under the unpermeabilized condition (Fig. 7C, compare profiles 4 and 5 in the bottom panel). These observations together indicate that binding of HCVpp to Huh7 cells may involve both receptor-independent and -dependent routes.

## Discussion

How HCV E1 and E2 work in conjunction to mediate fusion between cell and virus has been a long-standing question. Lavillette et al. characterized envelope variants with mutations in several membranotropic regions in E1 and E2 and found that certain mutations in the E1 fusion peptide-like domain may affect cell fusion and viral entry of HCV [63]. Although all four mutants they examined displayed strikingly reduced viral entry abilities, the Y276D and G282D mutants also showed a phenotype of impaired E1E2 incorporation into the virus. Thus, the decreased viral infectivity of those two mutants could not be attributed to their reduced viral entry capacity. The other two mutants, Y276F and G282A, exhibited inhibited fusion ability as well as an impaired entry capacity, indicating that the impaired viral entry of these two mutants was due to their reduced fusion ability. Nevertheless, the role of this fusion peptide-like domain in viral infection still remains to be determined. It is important to characterize more mutations in this region to provide better insights into how the E1 fusion peptide-like domain may act during HCV entry.

In the present study, we examined the effects of Ala and Asn substitutions for those residues conserved between HCV and E proteins of flaviviruses or the fusion proteins of paramyxoviruses on E1E2 properties. With the exception of the G267A mutant, all mutants showed reduced or marginally inhibited fusion ability compared to the WT E1E2 (Fig. 3). The decreased cell fusion ability of these mutants cannot be explained by a defect in total expression and/or cell surface expression of these mutants (Fig. 2), or by a defect in E2 binding to CD81 or E1-E2 interaction (Fig. 4). Since the CD81 binding of E2 is determined by three discontinuous elements brought by the folding of E2 [79,80], the comparable CD81 binding ability of E2 of these mutants to that of the WT protein (Fig. 4B) indicates that these mutations in E1 do not affect the overall structure of E2. Similar amounts of WT and mutant E1 proteins were coprecipitated by sCD81 through their association with E2 in this analysis (Fig. 4B) also implied that mutant E1 proteins effectively form a hetero-oligomer with E2. Importantly, similar levels of E1 and E2 were coprecipitated by an E2 conformation-dependent H53 MAb from WT and mutant plasmid transfections under nonreducing conditions (Fig. 4C), further indicating that mutations in this domain neither affect the overall conformation of E2 nor alter the assembly of E1 and E2 into a non-covalent heterodimer.

The partial loss of fusion ability of the G282A mutant observed here was also noted by Lavillette et al. [63]. With the exception of the G278A and D279N mutants, which exhibited reduced E1 and E2 incorporation into HCVpp virions, the other five mutations displayed a WT-like pattern of E1E2 incorporation into HCVpp virions (Fig. 6). Since pseudoparticles bearing each of the C272A, C281A, G282A, and G288A mutant proteins bound target Huh7 cells as effectively as did the WT HCVpp (Fig. 7), the impaired viral entry capacity of these four mutants (Fig. 5) was due to their reduced fusion ability (Fig. 3).

It was suggested that receptor-independent binding plays a crucial role during the early phase of virus attachment to facilitate retroviral particles reaching the specific receptor(s) and the subsequent entry process [78]. Although we could not distinguish the proportion of receptor-dependent and -independent routes in mediating the initial attachment of WT and mutant pseudotypes to Huh7 cells (Fig. 7), these analyses do show comparable binding of WT and mutant pseudotypes to cells. Therefore, the differential viral entry examined by these mutants should not be solely attributed to their difference in binding to host cells, but rather to their differential fusion ability.

Interestingly, we also noted that the severity of reductions in cell fusion and viral entry of the C272A, C281A, G282A, and G288A mutants paralleled the conserved nature of these residues among HCV genotypes. For instance, the Cys-272 and Cys-281 residues are highly conserved among the HCV and E proteins of flaviviruses (Fig. 1A), and mutations at these two residues greatly reduced the viral entry capacity (Fig. 5) and also inhibited cell fusion ability (Fig. 3). Gly-288 is less conserved than Gly-282 among HCV isolates (Fig. 1A), and a mutation at this residue displayed a smaller inhibitory effect on membrane fusion and viral entry compared to the Gly-282 mutant (Figs. 3, 5). Also, the C272A and C281A mutations exhibited more-significant inhibitory effects on viral entry than did the G282A and G288A mutants (Fig. 5).

It was shown that the fusion sequences from alphaviruses and flaviviruses include Cys residues for specific disulfide bridges which are essential for the folding stability of fusion peptides [81,82]. The observations that the E1 and E2 proteins of these two mutants did not migrated differentially from WT E1 and E2 proteins on both reducing and nonreducing gels (Fig. 2E) indicate that these two Cys residues are not involved in intra- or intermolecular disulfide bond formation. Therefore, the possibility that these two Cys residues are involved in the structural determinant of the fusion peptide-like domain is unlikely. These results collectively imply that these two highly conserved Cys residues located in this domain may directly play a crucial role in cell fusion and virus entry.

The observation that the G267A mutant exhibited higher viral entry ability compared to the WT proteins (Fig 5) correlates with its higher cell fusion ability compared to the WT E1E2 (Fig. 3). This characteristic of the G267A mutant is consistent with the fact that Gly-267 is not conserved in HCV genotype 2 (Fig. 1A). The increased cell fusion ability of this mutant might be due to a stabilization effect of an Ala substitution at this residue on E1's structure.

## Conclusion

Our study has implications in understanding the molecular basis of how the E1 fusion peptide-like domain acts during cell fusion and viral entry.

## Competing interests

The authors declare that they have no competing interests.

## Authors' contributions

HFL carried out all experiments, and participated in experimental design. CHH participated in the CD81-E1E2 protein pull-down assay and incorporation of E1 and E2 proteins into HIV-1 virons. LSA performed the control pseudotype binding and the flow cytometric analyses with the E2 MAb. CKC performed the sCD81 and E mutant construction. SSC conceived of the study, coordinated the work, and wrote the manuscript. All authors read and approved the final manuscript.
